# Case of Traumatic Tetanus, Occurring in the Norfolk and Norwich Hospital

**Published:** 1835-07-01

**Authors:** Archibald Dalrymple

**Affiliations:** Norwich.


*Case of Traumatic Tetanus, occurring in the Norfolk and Norwich Hospital.* Reported by Mr. Archibald Dalrymple, Norwich.

The extreme obscurity in which the subject of Tetanus is involved, both as regards its pathology and treatment, induces me to add one more case to the long list. I am the rather led to do so in the present instance, from the opportunity it afforded of a careful post-mortem examination, although I confess the appearances presented were neither new, nor tending to elucidate the general train of symptoms.

It is true we have yet reaped little benefit from the recorded histories of tetanus, and that the morbid lesions do not appear sufficiently marked to account for the horrible symptoms attending this malady: still less is there any plan of treatment which can be recommended on the score of general utility; since almost every article of the materia medica has been tried in vain, till ingenuity has been tortured to contrive some novelty in the remedial applications. It is, therefore, not with any view of suggesting improvements in the method of treating this disease that I detail the following case, but simply to place upon record the symptoms of the malady in conjunction with the morbid appearances presented by the great vital organs, a few hours after death.

John Adams, aged forty-four, a man of large frame and great muscular power, and also of intemperate and violent habits, was admitted, under the care of my father, into the Norfolk and Norwich Hospital, on the afternoon of Thursday, December 19th, 1833, with a severe injury of the right hand, produced by the limb being caught in a machine at work for cutting hay.

There was a compound fracture of the proximal phalanx of the index-finger, which hung only by the flexor tendons and a small portion of integument. The distal phalanges of the middle and ring fingers were completely separated from the second phalanges. There was a lacerated wound on the dorsal as well as on the palmar aspect; the integuments of the dorsum being drawn down over the fingers, like a reflected glove. There was no wound of the extensor tendons, nor was any articulation opened, except where the two last phalanges had been removed at the time of the accident. The wound in the palm was not deep, the palmar fascia not being divided. There was but little bleeding, two or three dorsal veins being alone divided. One or two branches of the inner cutaneous nerve were exposed. The man was drunk at the time of the accident.

The index-finger was removed, as well as one or two small splinters of bone at the point of fracture; the edges of the wounds were approximated as much as possible, and retained by means of adhesive plaster; a few strips of lint were placed over the wounded parts, and protected by a few light turns of a linen roller. Over these wet cloths were kept; the hand and forearm were raised; and an anodyne given. He passed a restless night, and in the morning became feverish and uneasy.

December 20th. He complained of much pain in the hand; his pulse was quick and full. There was some swelling of the lower part of the forearm, with very slight redness. Diaphoretics, with purgatives, were given, and low diet ordered. He continued much the same for the first three or four days, his bowels being carefully attended to, and his diet regulated.

On the morning of the fourth day, (Dec. 23d,) the first dressings were removed: the hand was a good deal swollen, and some little inflammation was apparent over the wrist; not, however, to any great extent. There had been no union of any part of the wound. A large meal poultice was ordered to the whole hand every five or six hours, and the same diet and medicines continued. He expressed himself as feeling languid, and unable to get any sleep, in spite of large anodynes. He went on thus for the next three days. The cuticle had begun to desquamate, and a slough, which had formed on the back of the hand, was beginning to separate: the discharge was very scanty and ill-conditioned. His pulse had become much reduced, and he was evidently a good deal pulled down by the treatment adopted. His skin had all along been soft and open; he had a clean and moist tongue, had less thirst, but was still restless. His hand was much easier.

On Thursday, the 26th, (a week after the accident,) his diet was bettered. He was ordered meat and broth, with half a pint of porter, daily.

On the 28th, there was an improvement in his general as well as local condition: there was more discharge, and of a better character, from the wound; the line of separation was more distinct, and a few small granulations were visible; the fingers also had assumed, but in a less degree, a healthier aspect. Bowels regular; skin moist. He expressed himself as feeling better, and as enjoying his diet.

On the 29th, things were progressing favourably; but he complained of a sense of stiffness in the muscles of the neck and in those of mastication, which he attributed to the cold air of the ward. His skin was moist, and his bowels freely open. In the evening, however, he was unable to open his mouth; his teeth were firmly closed; the masseteres and sterno-mastoidei were contracted, and their edges defined. He had pain down the neck and back, accompanied by convulsive action of the muscles along the spine. It was now evident that he laboured under tetanus-trismus with opisthotonos. Six grains of calomel and three grains of opium were given about eight o'clock, at bed-time, and one grain and a half of calomel, with one grain of opium, ordered every hour through the night.

On the morning of the 30th, he was much the same; the teeth *nearly* closed; had taken the pills every hour, combined with a little camphor; has had no sleep; perspires profusely. Bowels twice relieved; pulse soft, not very quick, and with little power; is sensible; wound looking much the same; the same medicines to be continued, and two drachms of the strong mercurial ointment ordered to be rubbed every hour along the spine.

A consultation was called at half-past eleven, A.M., at which the propriety of removing the limb was considered, but the unanimous opinion was against such a proceeding. It was determined to continue the same treatment, with the addition of a blister to the nape of the neck; the blistered surface to be subsequently dressed with some preparation of opium. The same diet, with wine, was continued; the hand to be still poulticed.

In the evening he expressed himself as feeling better, although there were no external signs of it. His pulse was fuller and quicker; his skin moist; bowels freely opened; mouth much in the same state. The muscles on the right side of the neck were more rigid than on the left; the abdominal muscles hard and tense; has had no sleep; has taken some nourishment; blister has drawn; is now lying on his right side, whilst in the morning he was half sitting up in bed, with his head retracted; has less convulsive action down his back, but says he is very sore on being touched; is now unable to swallow pills, and therefore takes forty minims of the Liq. Opii sed. at each dose; hand looking much the same.

On the 31st he was worse; had passed a bad night; the spasms were very frequent and distinctly marked, all of them having a tendency to draw the body backwards; there was this morning a convulsive action of the diaphragm, and of all the muscles of deglutition, whenever he attempted to swallow; part of the fluid generally returns, in doing which cough and viscid expectoration are produced. Even the approach of fluids to the mouth excites, but in a less degree, the same phenomena. The right sterno-mastoid is the more rigid of the two. The muscles of the abdomen were just the same; his skin very moist; bowels freely relieved. One drachm of the mercurial ointment has been rubbed in, and the same dose of sedative liquor given every hour or two, and also as much wine as the spasms would allow of. His gums are slightly affected by the mercury; pulse 150, smaller, and of less force; *says he feels much better*, and is able to open his mouth a *little* wider. What can be seen of his tongue appears to be white; prefers all drinks to be hot; the hand looks better; nearly all the slough on the dorsum of the hand being loose, was removed; the granulations are healthy, and the discharge rather more ample and creamy. The second phalanx of the ring finger is perfectly bare; poultices still continued; the blister is dressed with an ointment composed of one ounce of lard to one drachm and a half of powdered opium. He is now sitting up in bed, with the head drawn backwards; the posterior cervical muscles are very rigid. The mercury rubbed in is now reduced to one half.

This man died about six o'clock. In the interval between the morning visit and his death, he had been more or less convulsed. Every attempt to swallow brought on spasmodic action of the extensors of the back; of the muscles of the fauces and œsophagus and diaphragm, producing cough, followed by a viscid expectoration of mucus. During the attempts to swallow, the contortions of his features were frightful; the countenance assumed a leaden-black hue, and the muscles of the face were strikingly developed.

About three hours before his death his left side only appeared to be convulsed, and latterly the left hand and arm vibrated so rapidly as to resemble the pendulum of a clock; the injured arm scarcely moved. To the last he was sensible. The specific effect of the mercury was apparent on his gums, but neither it nor the opium for one instant relieved his symptoms.

The following were the appearances, after death, of the brain and spinal marrow; the fauces and œsophagus; the larynx and trachea; the heart and lungs.

*Brain.* Little more was observable than a remarkable dryness of the membranes, and especially of the arachnoid, which was so dry as to appear quite glazed. The brain itself was firm, very little appearance of arterial vascularity, but considerable venous congestion; almost an entire absence of those bloody points usually seen in cutting down to the centrum ovale.

*Spinal marrow.* Between the theca and arachnoid membrane there was from half an ounce to three quarters of an ounce of colourless fluid; between the arachnoid and pia mater of the spinal marrow in the lumbar region, there was also a little fluid, also colourless. In different places there were cloudy patches of the arachnoid very evident. There were signs of increased vascularity in the cervical region; these were less distinct in the upper part of the dorsal, but in the inferior part of the same region, and also in the whole of the lumbar, there were most unequivocal marks of recent inflammation. The whole of the 'cauda equina' was inflamed, and of a pinkish hue, just as is seen in inflammation of the sclerotic coat of the eye; the veins were congested and varicose; there was no visible deviation from the healthy texture of the part.

*Fauces* inflamed pretty generally, especially towards the pillars and amygdalæ; the papillæ at the base of the tongue were much enlarged and inflamed.

*Œsophagus* of a pinkish hue, and closely contracted; the inflammation less apparent, however, than any where else.

*The Larynx, Trachea,* and *Bronchi* were all similarly affected; the epiglottis was inflamed, but neither generally or intensely; the sacculi laryngis were considerably so. The larynx was generally inflamed, but in a less degree than the trachea, which, especially towards its division into the bronchi, was much reddened and inflamed; the bronchi were in a similar state, but became less so as they entered the roots of the lungs.

*Lungs* considerably congested at their posterior part; this partly arose from position. The free anterior edge of the right lung about its centre, was emphysematous over an area of two square inches, but this lesion was probably not of a recent date.

*Heart* soft and flabby; muscular structure easily lacerable between the finger and thumb. The coronary vein, at its opening, would admit the tip of the little finger. The lining membrane of the heart on the left side was of its usual colour; that on the right side was of a deep claret or maroon colour, not lost by washing repeatedly.

